# 

**Published:** 1893-01

**Authors:** 


### Southern Medical Record

A Monthly Journal of Medicine and Surgery.

VOL. XXIII. ATLANTA, GA., JANUARY, 1893. NO. 1.

EDITORS:

A. W. GRIGGS, M. D. WILLIS F. WESTMORELAND, M. D.

J. McFADDEN GASTON, M. D. DAN. H. HOWELL, M. D., Bus. Mgr.

Entered at the Postoffice at Atlanta, Ga., as Second-Class Matter.

S. W. Postell, Publisher, 23 S. Broad, Atlanta, Ga.

